# The mechanisms of ameliorating effect of a green tea polyphenol on diabetic nephropathy based on diacylglycerol kinase α

**DOI:** 10.1038/s41598-020-68716-6

**Published:** 2020-07-16

**Authors:** Daiki Hayashi, Liuqing Wang, Shuji Ueda, Minoru Yamanoue, Hitoshi Ashida, Yasuhito Shirai

**Affiliations:** Department of Applied Chemistry in Bioscience, Graduate School of Agricultural Science, Kobe University, Rokkodai-Cho 1-1, Nada-Ku, Kobe, 657-8501 Japan

**Keywords:** Kidney diseases, Diabetes complications, Nutrition

## Abstract

Significant efforts have been made to ameliorate diabetic nephropathy (DN) by inhibiting protein kinase C. However, these efforts have not been successful in human trials, suggesting that novel therapeutic strategies are required. Thus far, it has been reported that green tea polyphenol epigallocatechin gallate (EGCg) improved albuminuria in DN in a human trial. Our previous study revealed that activation of diacylglycerol kinase α (DGKα) plays a crucial role in the amelioration of DN and that EGCg activates DGKα. Here, we investigated whether and how DGKα contributes to the amelioration of DN upon stimulation by EGCg by using streptozotocin-induced type 1 diabetic model mice. Our results revealed that EGCg ameliorated albuminuria in DN through DGKα in vivo, and methylated EGCg, which has higher absorption in the plasma improved albuminuria in DN effectively. Additionally, we showed that c-Src mediated EGCg-induced DGKα translocation and colocalized with the 67 kDa laminin receptor, which is an EGCg receptor. Furthermore, EGCg attenuated the loss of podocytes in DN by preventing a decrease in focal adhesion under high glucose conditions. Our results indicate that the DGKα pathway is an attractive therapeutic target and that activating this pathway is a novel strategy for treating DN.

## Introduction

Diabetic nephropathy (DN) is one of the severe vascular complications of diabetes. It is classified by the lesion of glomerular based on the electron microscopy and light microscopy observation of the kidney biopsy^1^. DN causes glomerular filtration failure, leading to a need for dialysis therapy due to uremia, and DN has a strong association with albumin excretion. Diabetic kidney disease (DKD) is diagnosed by the albumin excretion and/or impaired glomerular filtration rate (GFR) in type 1 and type 2 diabetes^2^. Although there is a difference in the diagnosis, albuminuria is a typical symptom. Microvascular disorders in glomeruli due to polyol pathway activation, polyol glycation, and oxidation, caused by hyperglycemia, are known to cause DN. In addition to these causes, abnormal activation of conventional protein kinase C (cPKC) is also known as a cause of DN^3^. In hyperglycemia, the de novo synthesis of diacylglycerol (DAG) increases, and cPKC is abnormally activated since it is activated by DAG, resulting in diabetic vascular complications, including DN^4–7^. Indeed, the upregulation of the DAG-cPKC pathway in glomeruli has been observed under diabetic and high glucose conditions, and cPKC has been considered as one of the therapeutic targets for DN^8–10^. Among cPKCs, PKCβ is a primary target for ameliorating diabetic vascular complications, and a specific inhibitor of PKCβ has also been developed. A significant number of studies have implicated the ameliorating effect of this inhibitor on diabetic vascular complications, including DN^11–13^. However, the PKCβ-specific inhibitor has not passed a human clinical trial due to their insufficient efficiency in ameliorating diabetic vascular complications compared with the efficiency of the placebo^14^. These previous studies suggested that, for treating diabetic vascular complications, solely inhibiting PKCβ activity was insufficient.

Based on these facts, we have focused on DAG kinase (DGK) as a therapeutic target of DN. DGK is a lipid kinase that phosphorylates DAG and produces phosphatidic acid (PA)^15^. In other words, activation of DGK can suppress cPKC activation by reducing the amount of DAG. Thus far, Koya et al. reported that d-α-tocopherol (vitamin E, αToc) ameliorated DN by inhibiting PKC activity through DGK activation in rat glomeruli^16^. Mammalian DGKs consist of five types, which are divided into ten subtypes based on their structural features^17–19^. Thus far, we have shown that DGKα plays a critical role in the αToc-induced amelioration of DN through in vitro studies and in vivo studies using DGKα-deficient mice^20,21^. In addition, our previous study revealed that DGKα is highly expressed in the podocyte^22^. Podocyte is a terminally differentiated cell forming the slit membrane structure in the glomerulus, which functions as a filtration barrier^23^. In the DN condition, the collapse of the slit membrane structure and loss of podocyte cause filtration failure^24^. In our previous study, we found that αToc-induced DGKα activation prevents the loss of podocyte and the morphological changes in the slit membrane structure under diabetic conditions^21^. Additionally, we reported that oral administration of αToc ameliorated DN in mice. In other words, we found the possibility that the compound that activates DGKα can ameliorate DN^25^. However, the oral consumption of αToc did not ameliorate DN in a human trial^26^. Therefore, we tried to find a novel activator of DGKα to use as an agent for treating DN.

In our previous study, we revealed that the chroman ring structure of αToc is essential to activate DGKα^20^. Epigallocatechin gallate (EGCg) is a green tea polyphenol and has a chroman ring structure. Furthermore, we found that EGCg activated DGKα through the 67 kDa laminin receptor (67LR), which is known as an EGCg receptor, suggesting the possibility of using EGCg as an agent for treating DN^22^. Indeed, a significant DN ameliorating effect of EGCg was observed in a double-blind human trial, differed from that of the study on αToc and PKCβ inhibitors^27^. However, the mechanism of how EGCg ameliorated DN was unknown. Therefore, in the present study, we investigated the mechanism of the amelioration effect of EGCg on DN by focusing on 67LR and podocytes. To confirm the value of DGKα as a therapeutic target, we also sought to determine whether EGCg improved albuminuria in DN via DGKα by using the streptozotocin-induced type 1 diabetic model mice. The model is a well-established mouse model for studying DN, which shows glomerular basement membrane ticking and mesangial expansion as well as albuminuria^28^. In addition, to find a better treatment for DN, we evaluated the effect of epigallocatechin 3-(3″-O-methyl) gallate (EGCg3″Me), which is absorbed in higher amounts than EGCg, on symptoms of DN.

## Materials and methods

### Animals studies

All animal studies were approved by the Institutional Animal Care and Use Committee (permission number: 25-07-03) and performed according to the Kobe University Animal Experimentation Regulations. Wild type male C57BL/6NCrSlc mice were purchased from Japan SLC, Inc. and were housed in the Kobe University Life-Science Laboratory. We bred and used systemic DGKα-deficient C57BL/6N (DGKα^−/−^) mice that were gifted by Dr. Matthew K. Topham. All the mice were fed a DC-8 powder diet (CLEA Japan, Japan) and had free access to water. The protein concentration of DC-8 was 24.6% w/w. The sodium concentration of DC-8 was similar to CE-2 diet (0.33% w/w NaCl; CLEA Japan, Japan). The cages were maintained under a 14-h light and 10-h dark cycle at 23 ± 2 °C. To minimize the effect of body weight differences on the experiment, the body weight of all mice at five weeks of age was measured, and mice were assigned to respective experimental groups so that each group contained a similar total body weight. The mice in the same group were bred in a cage throughout the experiments (the number of mice (n) in each group was 4 or 5).

### Experimental design

Diabetes was induced in 6-week-old male mice through intraperitoneal administration of 50 mg/kg streptozotocin (STZ) in 20 mM citrate buffer for five consecutive days. It is known that STZ has direct toxicity on the kidney^29,30^. To minimize the toxicity of STZ on the kidney, we used a low dose of STZ (50 mg/kg) for producing diabetic model mice. The same volume of vehicle (12.5 ml/kg) was administered to the control group. We measured glucose levels in the blood drawn from mouse tails using Glu-test Sensor and Glu-test Every glucose meter (Sanwa Kagaku Kenkyuusho, Japan). After the final STZ administration, we fed the mice a DC-8 powder diet with or without various doses of powdered EGCg (Sunphenon EGCg-OP, Taiyo-Chemical Co. Ltd, Japan, purity ≥ 94%) or powdered EGCg3″Me (Nagara Science, Japan, purity ≥ 99%) throughout the experiments. After the last STZ administration and through the end of the experiment, we collected and measured the volume of urine from each group, which were subjected to fasting conditions, using a metabolic gauge (Tecniplast, Italy) for 8 h during the light period once a week. We measured body weight and blood glucose levels from all the mice before/after STZ administration and following the urine collection. At the end of the experiments, all the mice were anesthetized, and then, kidney was collected. Urine albumin excretion measurement was performed by following the method we published previously^25^.

### Immunoelectron microscopy

The male C57BL/6N mice were sacrificed with cervical dislocation, and a fixing solution (4% PFA and 0.2% glutaraldehyde in 0.1 M phosphate buffer) was perfused through the left ventricle. The kidney was removed and cut into 2 mm cubes. The cubes were further fixed and were dehydrated with ethanol and infiltrated in LR white resin. We polymerized the cubes with ultraviolet light for 48 h at − 20 °C and sliced the cubes into sections with a thickness of 100 nm using an ultramicrotome (Leica, Germany). We stained the sections using the 67LR antibody (N2C3, Gene Tex Inc., USA) as a primary antibody and observed the sections through a transmission electron microscope.

### Immunofluorescent staining of kidney

Immunofluorescent staining of kidney was performed by following the method we published previously using the nephrin antibody (GP-N2, PROGEN Biotechnik, Germany) as the primary antibody ^25^.

### Culture and differentiation of conditionally immortalized human podocytes

Conditionally immortalized human podocytes^31^ were cultured in RPMI-1640 medium supplemented with 10% fetal bovine serum, 1 × ITS (Insulin, Transferrin, and Selenite) liquid media supplement (Sigma-Aldrich, USA), and 1 × penicillin–streptomycin solution (Wako, Japan) in a humidified atmosphere containing 5% CO_2_ at 33 °C. To induce the cell to differentiate, the temperature was increased to 37 °C, and the cells were cultured for 14 days with the medium refreshed once every 2 days.

### Observation of GFP-DGKα translocation

Observation of GFP-DGKα translocation and calculation of translocation-rate were performed following the previous method ^22^. In short, DDT1-MF2 cell or undifferentiated conditionally immortalized human podocyte on glass-bottom dish has transfected the plasmid expressing GFP-DGKα. Twenty-four hours after transfection, the various concentration of EGCg or EGCg3″Me was gently added into glass-bottom dish under observation by confocal microscopy for 3 min.

### Detection of tyrosine phosphorylation and immunoprecipitation

Various plasmids were transfected into DDT1-MF2 cells on a 60 mm culture dish. The cells were lysed in lysis buffer (20 mM Tris–HCl at pH 7.4, 1 mM EDTA, 1 mM EGTA, 1 mM MgCl_2_, 1 mM phenylmethylsulfonyl fluoride, 1 μg/ml leupeptin, 1 mM NaF, and 1 mM Na_3_VO_4_) 24 h after cell transfection. When the cell was stimulated with EGCg, the medium was changed to a medium containing 300 μM EGCg and incubated for 15 min before the cells were harvested. The lysate was subjected to immunoprecipitation using a GFP antibody and protein G sepharose (GE Healthcare, USA) or FLAG-resin (Wako, Japan). The precipitated sample was subjected to western blotting following SDS-PAGE.

### Evaluation of focal adhesion

The differentiated human podocytes were plated in glass-bottom dishes and cultured for 72 h in normal growth medium (control), RPMI 1,640 medium containing 5.5 mM glucose and 24.5 mM mannitol (mannitol), RPMI 1,640 medium containing 30 mM glucose (high glucose) or RPMI 1640 medium containing 30 mM glucose and 50 mM EGCg (high glucose + EGCg). The cells were fixed with 4% PFA and stained with paxillin antibody (ab32084, Abcam, UK) and rhodamine-phalloidin. The stained cells were observed through a confocal laser microscope, and the focal adhesions stained with paxillin in randomly selected 25 μm^2^ area were counted.

### Statistical analysis

All error bars show the standard error of the mean (SEM). One-way ANOVA followed by Tukey–Kramer’s multiple comparison test was carried out to determine significant differences among the groups in the experiments that had more than three groups. The number of groups subjected to the test was indicated in the legends of figures. Student's t-test was carried out for experiments with two groups. Statistical analysis was performed using BellCurve for Excel version 3.20. A *p*-value of less than 0.05 was considered to be significant.

## Results

### The effects of the oral administration of EGCg on STZ-induced diabetes in mice

First, to confirm that the oral administration of EGCg ameliorated DN in mice, we conducted an animal experiment using STZ-induced diabetic model mice. As shown in Supplemental Fig. 1, the oral administration of diet containing 1% EGCg significantly ameliorated the increase in urine albumin excretion and in urine volume, which are symptoms of DN, without significant effect on the blood glucose levels and body weight. These results confirmed that the oral administration of EGCg ameliorated DN in the mice. Furthermore, we tested diets containing 0.05, 0.1, and 0.5% EGCg. The results showed dose-dependent amelioration of DN, and even the diet containing 0.05% EGCg showed a tendency to ameliorate DN (Supplemental Fig. 2).

### DGKα contributes to the effect of EGCg on DN

**Figure 1:**
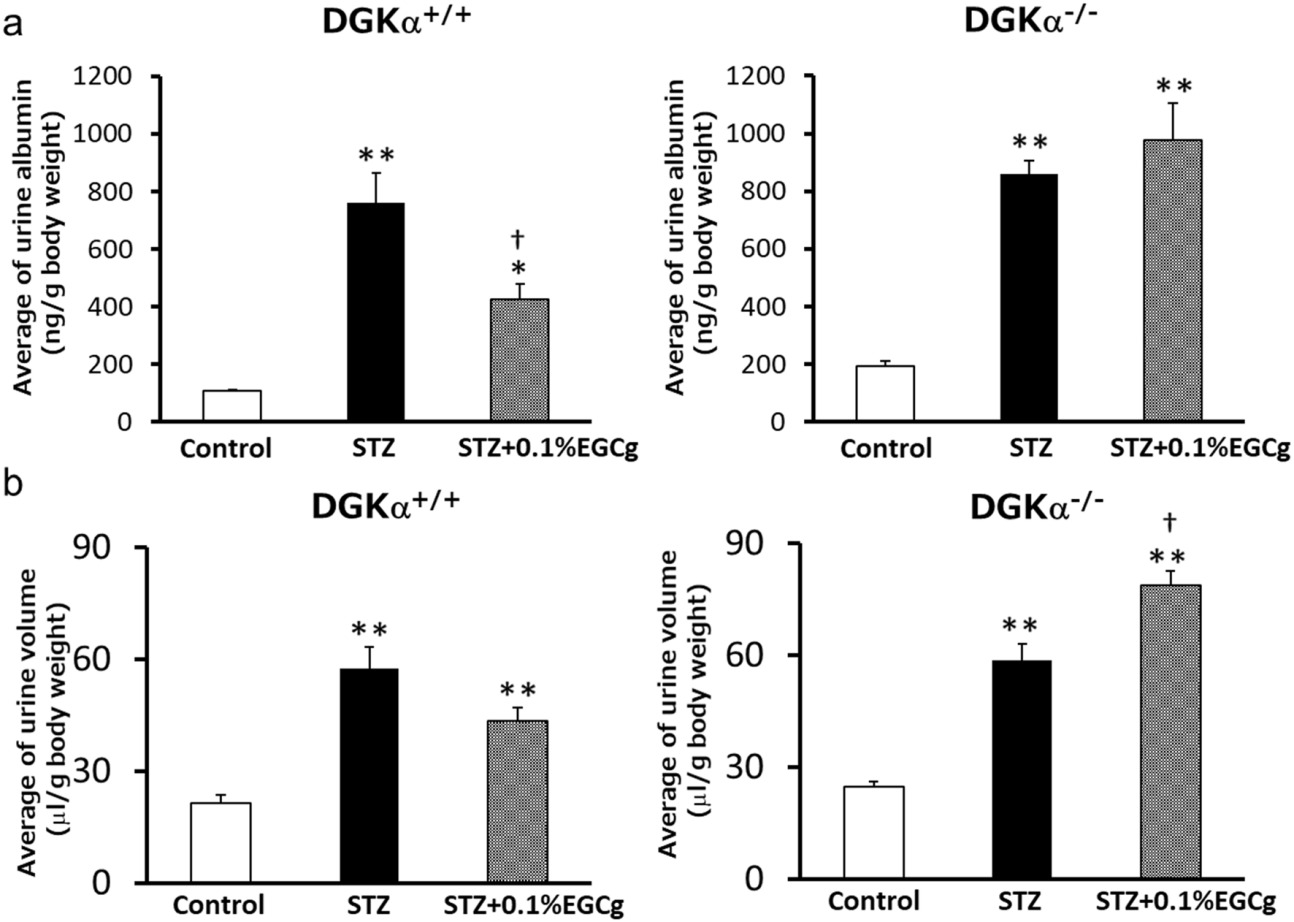
**The effects of oral administration of 0.1% EGCg on DGKα**^**+/+**^ **and DGKα**^**−/−**^ **mice**. The average urine albumin amount (**a**) and urine volume (**b**) from DGKα^+/+^ and DGKα^−/−^ mice. The number of mice in every group was n = 4. The values are ± SE. *p < 0.05, *p < 0.01 compared with control. †*p* < 0.05 compared with STZ. One-way ANOVA followed by Tukey–Kramer’s test between 3 groups was used for evaluating statistical significance.

To investigate whether DGKα is involved in the effects of EGCg on DN, we tested the effects of EGCg on systemic DGKα-deficient mice (DGKα^−/−^) with STZ-induced diabetes. In this experiment, we fed the diet containing 0.1% EGCg based on the experiment shown in Supplemental Fig. 2 and measured the urine albumin excretion and urine volume. The changes in blood glucose level and in body weight in each group are shown in Supplemental Fig. 3. Notably, EGCg administration did not improve the blood glucose level itself. The urine albumin amount was increased in both the DGKα^+/+^ and DGKα^−/−^ mice by STZ treatment. The increase was significantly suppressed by 0.1% EGCg administration in the DGKα^+/+^ mice but not in the DGKα^−/−^ mice (Fig. 1a). The urine albumin to creatinine ratio was also improved in the DGKα^+/+^ mice but not in the DGKα^−/−^ mice (Supplemental Fig. 4). Importantly, the result was consistent with the total albumin secretion showed in Fig. 1a. Therefore, we evaluated only the total albumin secretion for the rest of the study. A urine volume increase was also observed in both the DGKα^+/+^ and DGKα^−/−^ mice, but 0.1% EGCg administration showed a tendency to reduce the volume in the DGKα^+/+^ mice, although the difference was not significant (Fig. 1b). On the one hand, the urine volume in the DGKα^−/−^ mice became even higher upon EGCg administration. In the experiment, EGCg did not show any positive effect on DN in the DGKα^−/−^ mice, which indicated that DGKα dominantly contributes to the ameliorating effect of EGCg on DN in vivo.

### EGCg3″Me effectively ameliorates DN

**Figure 2:**
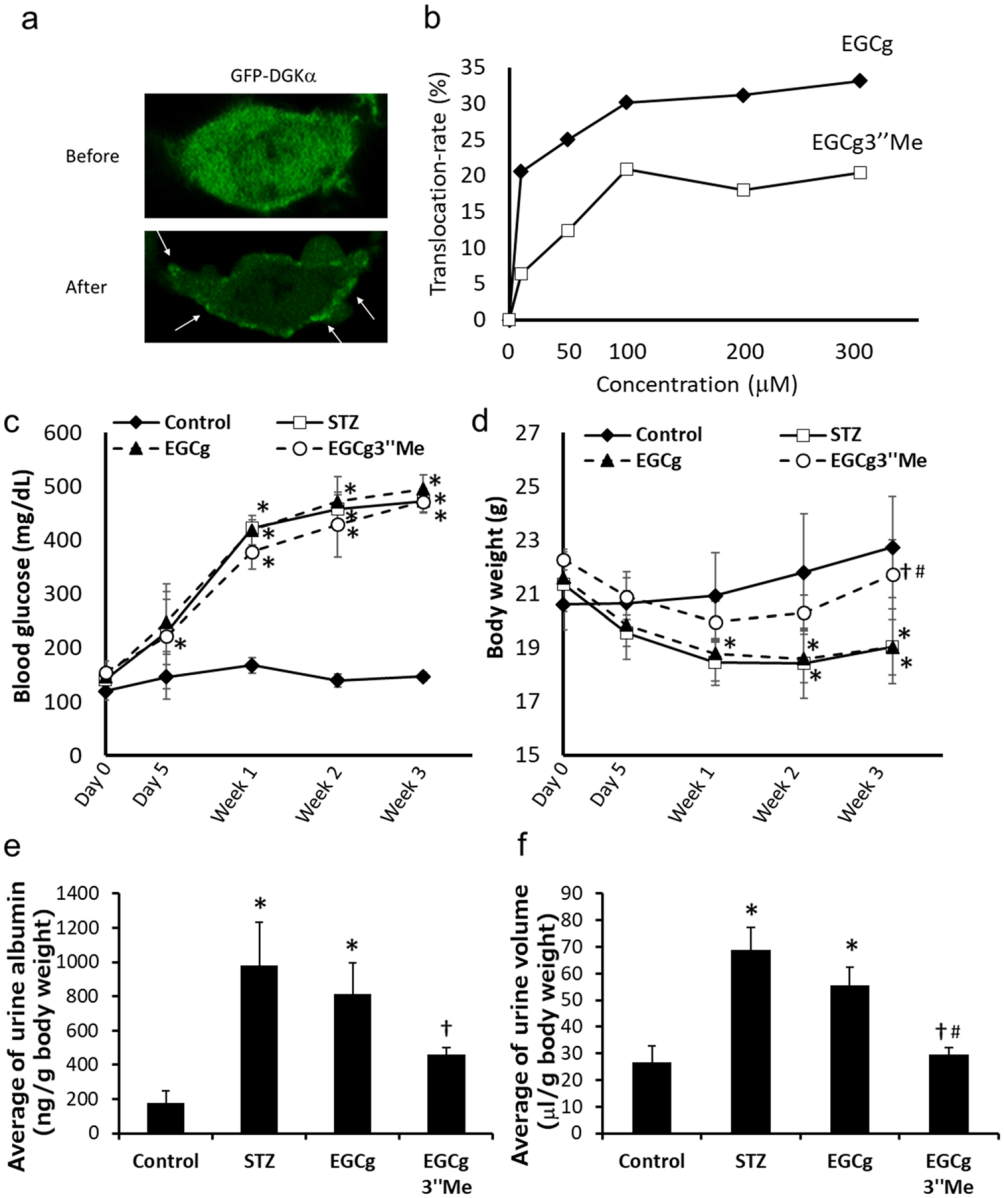
**The effects of EGCg3″Me on DN in mice.** GFP-DGKα overexpressed DDT1-MF2 cell was stimulated with 200 μM EGCg3′’Me for 3 min under confocal scanning microscopy (**a**). Translocation-rate of GFP-DGKα induced by 1, 10, 50, 100, 200, and 300 μM EGCg or EGCg3′’Me were measured (**b**). Blood glucose level (**c**) and body weight (**d**) of mice from each group were measured before/after STZ administration (day 0 and 5) and every week until week 3. Urine albumin amount (**e**) and urine volume (**f**) of mice from each group were measured every week after STZ treatment until week 3. The graph shows the average of them. The number of mice in every group was n = 4–5. The values were ± SE. *p < 0.05, compared with control. †*p* < 0.05 compared with STZ. #*p* < 0.05 compared with EGCg. One-way ANOVA followed by Tukey–Kramer’s test between 4 groups was used for evaluating statistical significance.

It is known that the absorption of EGCg in plasma is low^32^. EGCg3″Me has an *O*-methyl group on the galloyl moiety and compared with the amount of EGCg, higher amounts of EGCg3″Me are absorbed in plasma^33,34^. We hypothesized that EGCg3″Me can ameliorate DN more effectively than EGCg does. Then, we compared the effects of EGCg3″Me and EGCg on the symptoms of DN to determine the possibility of creating a more effective DN treatment agent. Before the experiments, we checked the effect of EGCg3″Me on the DGKα translocation to the plasma membrane, which is a hallmark of DGKα activation, using DDT1-MF2 cells expressing GFP-DGKα. Consequently, we confirmed that EGCg3″Me induced the translocation of DGKα (Fig. 2a). Additionally, EGCg3″Me induced translocation in a dose-dependent manner, but the translocation ability was lower than that of EGCg (Fig. 2b). Next, we fed STZ-induced diabetic mice a diet containing 0.05% EGCg or EGCg3″Me and monitored albuminuria and increase of urine volume in DN until week 3. Also, we measured the concentration of EGCg and EGCg3″Me in their plasma. The concentration of EGCg3″Me in the plasma of EGCg3″Me-treated mouse was higher than that of EGCg in the plasma of EGCg-treated mouse (Supplemental Fig. 5). It is known that EGCg is excreted in the urine through the kidney^35,36^. Therefore, we assumed that the EGCg and EGCg3″Me level in the plasma and kidney were correlated.

There was no significant effect of EGCg or EGCg3″Me on fasting blood glucose levels (Fig. 2c). However, EGCg3″Me significantly prevented body weight loss (Fig. 2d). It is well known that diabetes causes body weight loss because of the loss of availability of glucose as energy, resulting from insulin tolerance or decrease of insulin secretion. Therefore, there is a possibility that EGCg3″Me improved the symptoms of diabetes itself.

More interestingly, the oral administration of 0.05% EGCg did not show a significant effect on the increase in urinary albumin amount. However, 0.05% EGCg3″Me administration normalized the urinary albumin amount (Fig. 2e). Oral administration of 0.05% EGCg significantly suppressed the STZ-induced urine volume increase, and notably, oral administration of 0.05% EGCg3″Me remarkably suppressed the increase compared with that of the EGCg group (Fig. 2f). We normalized the total albumin amount and urine volume by the body weight of mice because the difference in body weight might affect the water consumption and the basal urine volume. However, as shown in Fig. 2d, the body weight of mice treated with EGCg3″Me increased by 14% at week 3 compared to that of mice treated with EGCg. It may affect the calculation of the albumin amount and urine volume. Notably, the albumin amount and urine volume from mice treated EGCg3″Me decreased by 44% and 47%, respectively, compared to that from mice treated EGCg (Fig. 2e,f). Therefore, we assumed that the effect of body weight increase in EGCg3″Me-treated mice on the calculation should be ignorable. These results suggested that agents that activate DGKα and are absorbed in high amounts in plasma, such as EGCg3″Me, can be the better treatments for DN.

### The mechanisms of EGCg-induced DGKα activation

**Figure 3:**
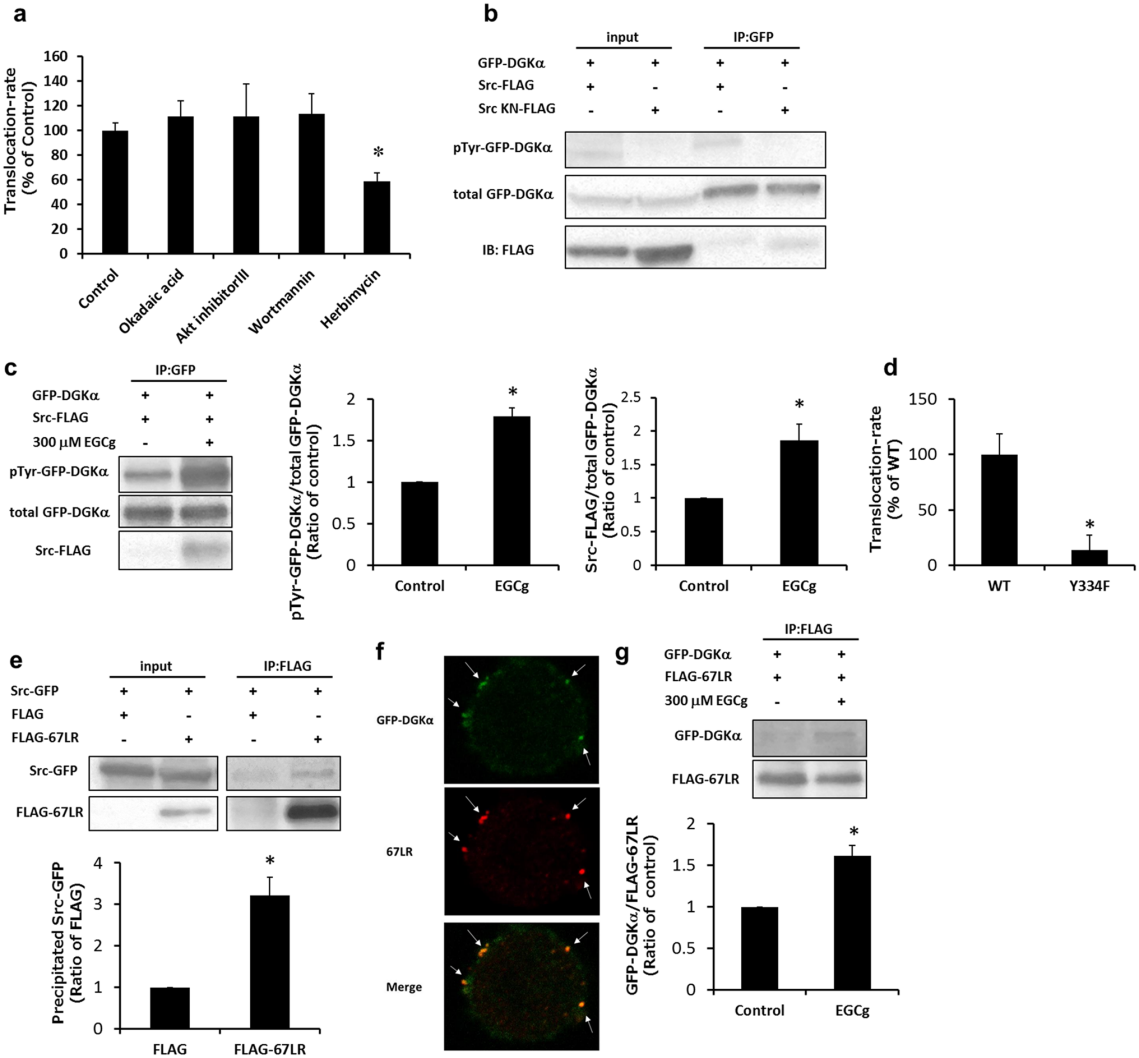
**The investigation of the signaling pathway of EGCg-induced DGKα activation.** DDT1-MF2 cell was pretreated with 1 μM okadaic acid, 10 μM Akt inhibitor III, 2 μM Wortmannin, or 2 μM Herbimycin for 30 min, and translocation of GFP-DGKα induced by 200 μM EGCg was observed (**a**). The equal volume of DMSO was used as the control. GFP-DGKα was co-expressed with c-Src-FLAG or c-Src KN-FLAG in DDT1-MF2 cell, and the cell lysate was immunoprecipitated using GFP antibody. The tyrosine phosphorylation was detected using the 4G10 antibody (**b**). The tyrosine phosphorylation of precipitated GFP-DGKα and the coprecipitated c-Src with or without stimulation of 300 μM EGCg for 15 min were detected (**c**). The translocation of GFP-DGKα (WT) or GFP-DGKα Y334F induced by 200 μM EGCg were observed and calculated translocation rate (**d**). Coprecipitation of FLAG-67LR and c-Src-GFP was detected immunoprecipitation using FLAG antibody-conjugated resin (**e**). The localization of GFP-DGKα and endogenous 67LR after stimulation with 200 μM EGCg for 3 min (**f**). Arrows indicate the colocalization of DGKα and 67LR on the plasma membrane. The coprecipitation of GFP-DGKα and FLAG-67LR with or without stimulation of 300 μM EGCg for 15 min (**g**). The intensity of each band of the western blot was analyzed by Image J. The values were ± SE. *p < 0.05 compared with control. One-way ANOVA followed by Tukey–Kramer’s test between 5 groups was used for evaluating statistical significance for the experiment (**a**), and Student’s t-test was used for the other experiments. Full-length blots are presented in Supplemental Figs. 6 to 9.

In a previous study, we revealed that EGCg activated DGKα via 67LR^22^. To elucidate how EGCg ameliorates DN through DGKα activation, we investigated the signaling pathway, focusing on 67LR. It was reported that protein phosphatase 2A (PP2A) and Akt mediated EGCg-67LR signaling^37,38^. In addition, it has been suggested that phosphoinositide 3-kinase (PI3K) and Src family tyrosine kinases are involved in DGKα activation^20,39,40^. Therefore, we determined the effects of the respective inhibitors on EGCg-induced DGKα translocation. Pretreatment with okadaic acid (a PP2A inhibitor), Akt inhibitor III and wortmannin (a PI3K inhibitor) did not show any significant effects on the translocation of DGKα. On the one hand, herbimycin (a Src family tyrosine kinase inhibitor) significantly reduced the translocation of DGKα by 50% (Fig. 3a). On the basis of these results, we sought to determine whether DGKα is phosphorylated by c-Src. Phosphorylated GFP-DGKα was detected when it was co-expressed with FLAG-tagged c-Src but not with c-Src kinase negative (KN) mutant (Fig. 3b and Supplemental Fig. 6). Additionally, c-Src or c-Src KN was coprecipitated with GFP-DGKα. Then, we determined whether the phosphorylation level of DGKα was increased by EGCg stimulation. We found that tyrosine phosphorylation was significantly enhanced and that the amount of coprecipitated c-Src increased upon EGCg stimulation (Fig. 3c and Supplemental Fig. 7). Thus far, it has been reported that phosphorylation of 334th tyrosine in DGKα (335th tyrosine in human DGKα) by c-Src is critical for the DGKα activation and translocation^20,40^. As shown in Fig. 3d, the Y334F mutant which mimics the unphosphorylated form of DGKα showed a significantly lower translocation-rate in response to EGCg stimulation than the wild-type DGKα (WT). These results suggested that EGCg stimulation-induced direct phosphorylation at 334th tyrosine in DGKα is necessary for its activation and translocation.

Next, to investigate the relationship between c-Src and 67LR, we carried out immunoprecipitation. Interestingly, coprecipitation of c-Src and 67LR was detected in the FLAG antibody precipitation fraction (Fig. 3e and Supplemental Fig. 8). Our previous study suggested that DGKα is translocated toward 67LR on the plasma membrane upon EGCg stimulation^22^. Similarly, we confirmed the colocalization of GFP-DGKα and the stained endogenous 67LR on the plasma membrane (Fig. 3f). Additionally, we investigated the binding between DGKα and 67LR by immunoprecipitation with DDT1-MF2 cells co-expressing GFP-DGKα and FLAG-67LR. We detected a weak signal indicating coprecipitated DGKα without EGCg stimulation; however, importantly, EGCg stimulation significantly enhanced the amount of coprecipitated DGKα (Fig. 3g and Supplemental Fig. 9). These results indicated that EGCg activated DGKα via 67LR and c-Src and that activated DGKα interacted with 67LR on the plasma membrane.

### EGCg prevents podocyte loss in DN via DGKα

**Figure 4:**
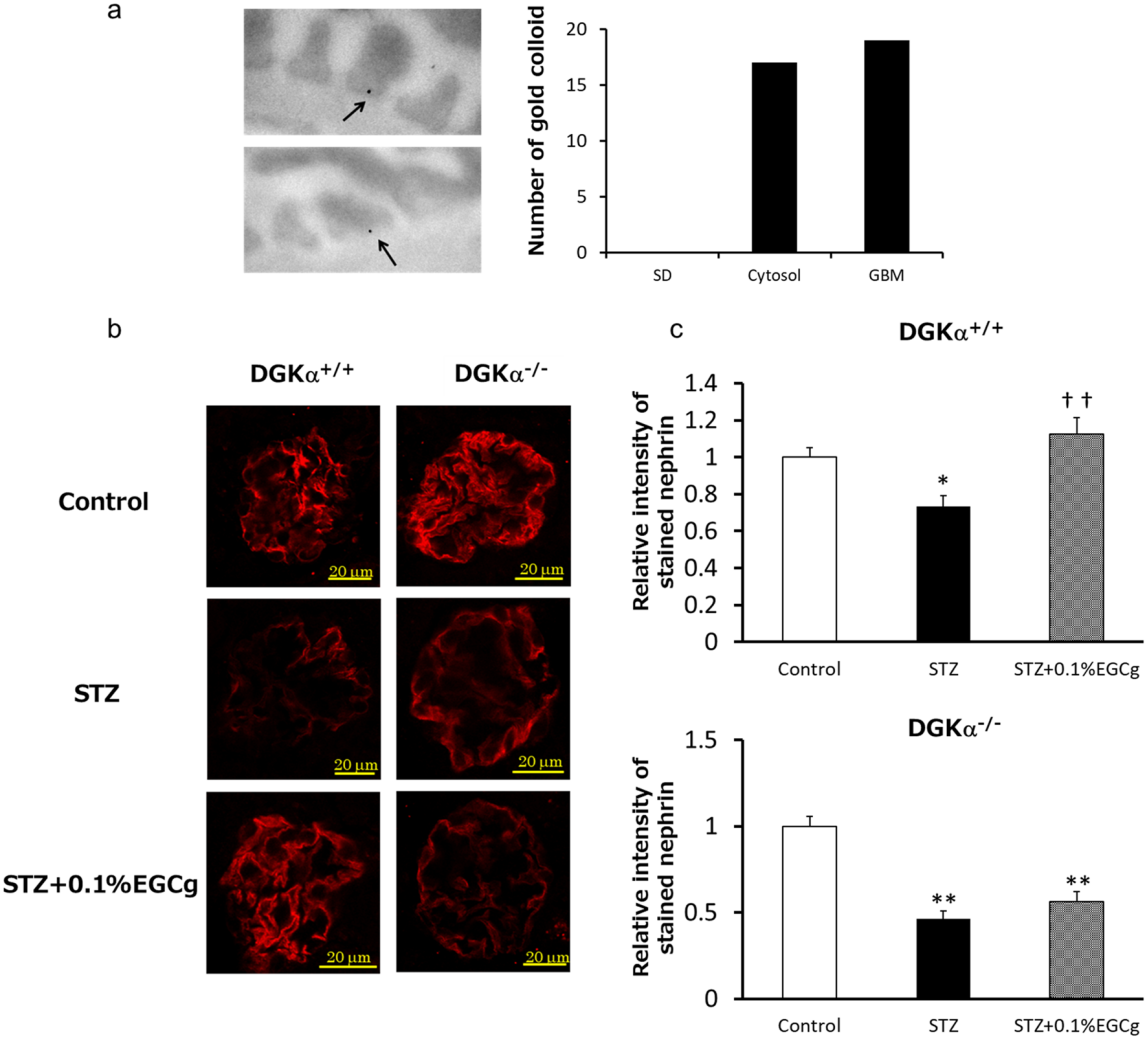
**Localization of 67LR in FP and the effects of EGCg on podocyte loss.** Immunoelectron microscopy observation of mice kidneys was performed by using the 67LR antibody as a primally antibody and gold colloid-conjugated anti-rabbit antibody as a secondary antibody. Arrows pointed at observed gold colloid in FPs. The number of gold colloids was counted and categorized with its localization (**a**). The podocyte loss in the kidney from DGKα^+/+^ and DGKα^−/−^ mice at week 6 after STZ administration was evaluated by immunofluorescent staining of nephrin (**b**) and the fluorescence intensity was analyzed (**c**). The values are ± SE. *p < 0.05, **p < 0.01 compared with control. ††*p* < 0.01 compared with STZ. One-way ANOVA followed by Tukey–Kramer’s test between 3 groups was used for evaluating statistical significance.

In our previous study, we revealed that both DGKα and 67LR are expressed in podocytes which is a terminally differentiated cell forming slit membrane structure as a filtration barrier in the glomerulus^22,23^. Podocytes interdigitated cell swelling called the foot process (FP) aligning on the glomerular basement membrane (GBM) via slit diaphragm (SD) to form the slit membrane structure^23^. The electron microscopy image of the slit membrane structure is shown in Supplemental Fig. 10. Firstly, we determined the precise localization of 67LR in the podocytes with immunoelectron microscopy to try to speculate the physiological meaning of DGKα translocation toward 67LR on the plasma membrane during the amelioration of DN by EGCg. The gold colloids that revealed the localization of 67LR were observed in the FP on the adhesive surface on the GBM and in the cytosol but not on the SD (Fig. 4a). Slit membrane formation collapses due to podocyte loss in DN, leading to filtration failure^41^. Therefore, we hypothesized that EGCg-induced DGKα activation and translocation strengthen the adhesion of the podocytes based on the result that 67LR exists on the adhesive surface on GBM. Then, to evaluate podocyte loss in vivo, in week 6, we used immunofluorescence stained nephrin, a marker of podocytes, in the kidneys of the STZ-treated diabetic DGKα^+/+^ and DGKα^−/−^ mice. The signal from the stained nephrin became weak in the STZ-treated DGKα^+/+^ and DGKα^−/−^ mice compared with those respective controls, indicating that podocyte loss had occurred because of DN (Fig. 4b upper and middle panels). The oral administration of 0.1% EGCg remarkably rescued the attenuation of the staining in the DGKα^+/+^ mice glomeruli, but this tendency was not observed in the DGKα^−/−^ mice glomeruli (Fig. 4b lower panel). Indeed, the fluorescence intensity of the stained nephrin in diabetic mice was significantly enhanced by EGCg administration in the DGKα^+/+^ mice but not in the DGKα^−/−^ mice (Fig. 4c). These results suggested that EGCg-induced DGKα activation prevents podocyte loss in DN.

### EGCg enhances adhesion by increasing the number of focal adhesions in human podocytes

**Figure 5:**
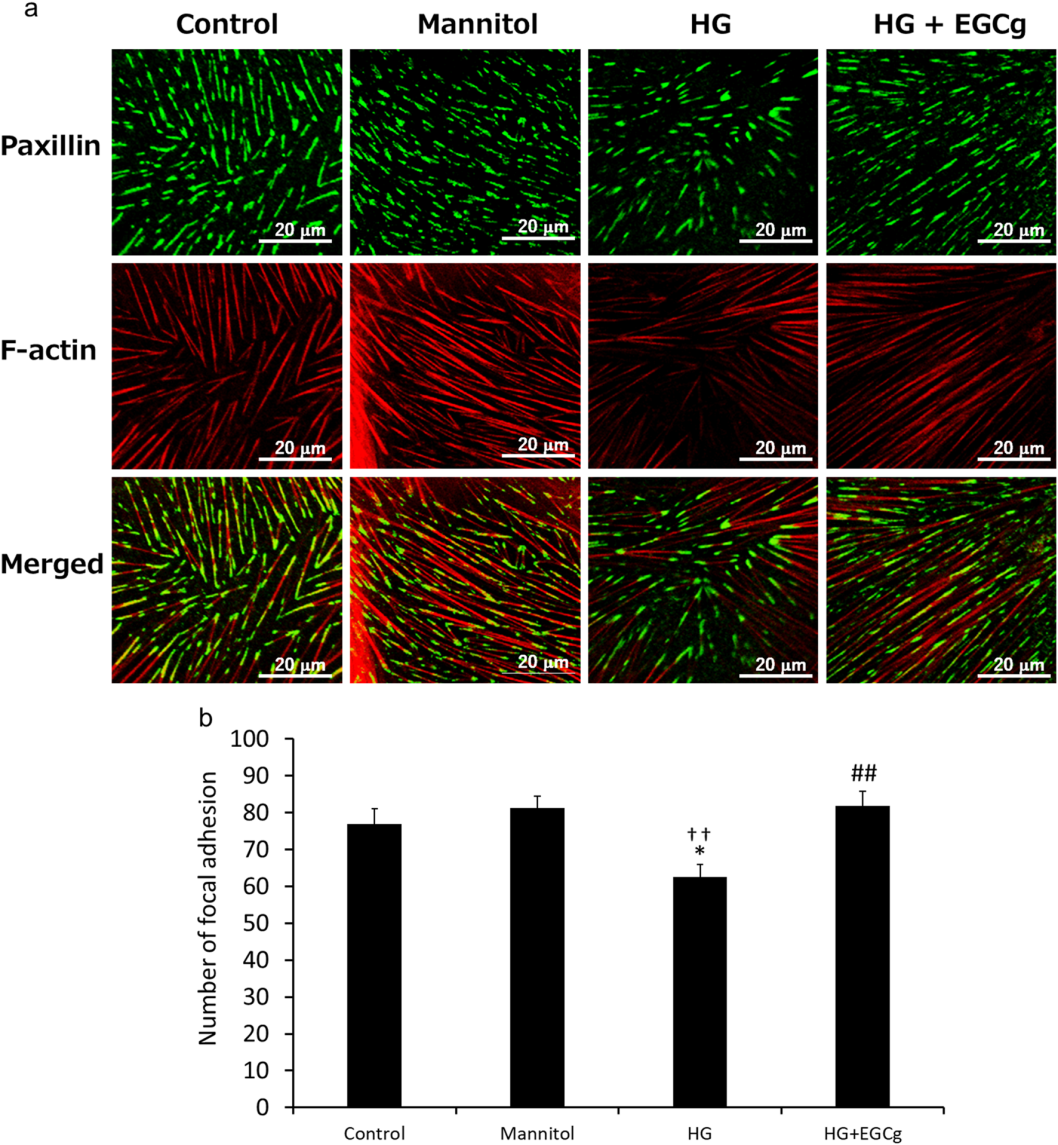
**The effect of EGCg on the high glucose-induced decrease in the number of focal adhesions in the differentiated podocyte.** The typical image of stained paxillin and F-actin (**a**). The number of focal adhesions in a radius of 25 μm^2^ was counted (**b**). HG: High glucose. The values are ± SE. *p < 0.05, compared with control. ††*p* < 0.01 compared with Mannitol. ##*p* < 0.01 compared with HG. One-way ANOVA followed by Tukey–Kramer’s test between 4 groups was used for evaluating statistical significance.

Finally, we used an immortalized human podocyte cell line, in which we confirmed the translocation of GFP-DGKα by EGCg (Supplemental Fig. 11a), to investigate how EGCg protects against the loss of podocytes in DN. We first determined cell differentiation by the increase of cell sizes, and specific nephrin staining at the terminus of F-actin (Supplemental Fig. 11b). We next determined the number of focal adhesions of the differentiated podocyte in the presence or absence of EGCg, based on the number of stained paxillin observed at the terminus of F-actin on the bottom of the cell. Mannitol was used as a control of osmotic pressure. The density of the paxillin and F-actin decreased under high glucose (HG) conditions (30 mM glucose) compared to their density in the controls (Fig. 5a left three rows). However, the decrease of the density of paxillin and F-actin fluorescence under the HG conditions was rescued by the presence of 50 μM EGCg (Fig. 5a right row). Indeed, the amount of stained paxillin significantly decreased under HG conditions, but EGCg attenuated the decrease (Fig. 5b). These results indicated that EGCg contributed to the prevention of focal adhesion decreases induced by HG conditions to protect podocyte loss.

## Discussion

In our previous study, we reported that green tea polyphenols, especially EGCg, could be agents for ameliorating DN through DGKα activation, and Borges et al*.* reported a significant ameliorating effect of EGCg on DN in a human trial^22,27^. In the present study, for the first time, we clearly showed that oral administration of EGCg ameliorated DN through DGKα in vivo.

DGKα is expressed ubiquitously and contributes to various biological processes. It is well known that DGKα is involved in the T cell anergy in vivo^42^. Also, it was reported that DGKα is highly expressed in melanoma cells and suppresses the apoptosis of melanoma^43^. Indeed, it was reported that the inhibition of DGKα activity enhances the apoptosis of melanoma^44^. Therefore, there is a possibility that systemic activation of DGKα may affect these biological functions of DGKα, and it might be the side effect of DGKα activation for treating DN, although the impact should be investigated in the future.

Additionally, EGCg3″Me, which is absorbed in plasma in higher amounts than is EGCg, ameliorated DN more effectively than EGCg. It has been demonstrated that catechins, including EGCg, have a preventive effect on high blood glucose^45,46^. In this study, oral administration of EGCg and EGCg3″Me did not improve fasting blood glucose level (Fig. 2c). However, the mice treated EGCg3″Me showed an improvement in body weight loss in diabetes (Fig. 2d). This fact suggested that EGCg3″Me showed some positive effect on diabetes itself. Therefore, we are hypothesizing that EGCg3″Me affected the casual blood glucose level, and we plan to evaluate this in a future study.

Although EGCg3″Me effectively improved albuminuria and increase of urine volume in DN, its activation efficiency on DGKα was almost one-half that of EGCg. It has been reported that EGCg3″Me also binds to 67LR, but its affinity to 67LR was relatively low compared with that of EGCg, suggesting that the affinity affected the activation efficiency^47^. However, the results from an in vivo study suggested that the stability and absorption of EGCg3″Me in plasma considerably compensated for the activation efficiency of DGKα. In other words, it was suggested that the balance of the activation efficiency with the stability and absorption in plasma is vital to the amelioration of DN by DGKα activation. Additionally, it is well known that oxidative stress is one of the causes of DN and that EGCg is an antioxidant^48,49^. In this study, it was assumed that EGCg functions as not only a DGKα activator but also an antioxidant. However, EGCg did not show any ameliorating effects on DN in the DGKα^−/−^ mice, indicating that even though the antioxidative effect of EGCg was supportive, it was not sufficient to ameliorate DN.

Furthermore, we revealed the mechanisms of the ameliorating effect of EGCg on DN by focusing on 67LR and podocytes. c-Src was involved in the EGCg-induced DGKα activation via 67LR. The 67LR is considered to act as a homo or heterodimer of the 37 kDa precursor, but the detailed mechanisms of how to transmit the signals are unclear^50^. To date, Akt and PP2A have been shown to be downstream of 67LR and EGCg^37,38^. However, these inhibitors did not affect the EGCg-induced translocation of DGKα. Our results indicated that c-Src directly phosphorylated and activated DGKα by responding to EGCg stimulation. Interestingly, we found that c-Src interacted with 67LR. Moreover, the results that DGKα translocated to 67LR on the plasma membrane and bound 67LR suggested that DGKα, c-Src, and 67LR formed a complex through EGCg stimulation.

To date, cPKC, especially PKCβ, has been one of the primary targets for DN. However, the specific PKCβ inhibitor failed clinical trials. In the present study, although DGKα is an indirect suppressor of the abnormal activation of PKCβ, EGCg-induced DGKα activation ameliorated DN, suggesting another mechanism contributing to the amelioration in the case of EGCg. In addition to PKCβ, it has been reported that abnormal activation of PKCα which is one of cPKCs exacerbates DN by enhancing transforming growth factor-β (TGF-β) and vascular endothelial growth factor (VEGF) signaling pathways in podocytes^51–54^. Additionally, it was suggested that activation of PKCα collapsed the morphology of FP by promoting endocytosis of nephrin, which is critical for adhesion between FP in DN^55^. Since DGKα can inhibit cPKC activity by reducing the amount of DAG, DGK can inhibit not only PKCβ but also PKCα at the same time. That seems to be the reason why EGCg improved DN in humans. It can be one of the advantages of DGKα activation instead of directly inhibiting PKCβ activity.

**Figure 6:**
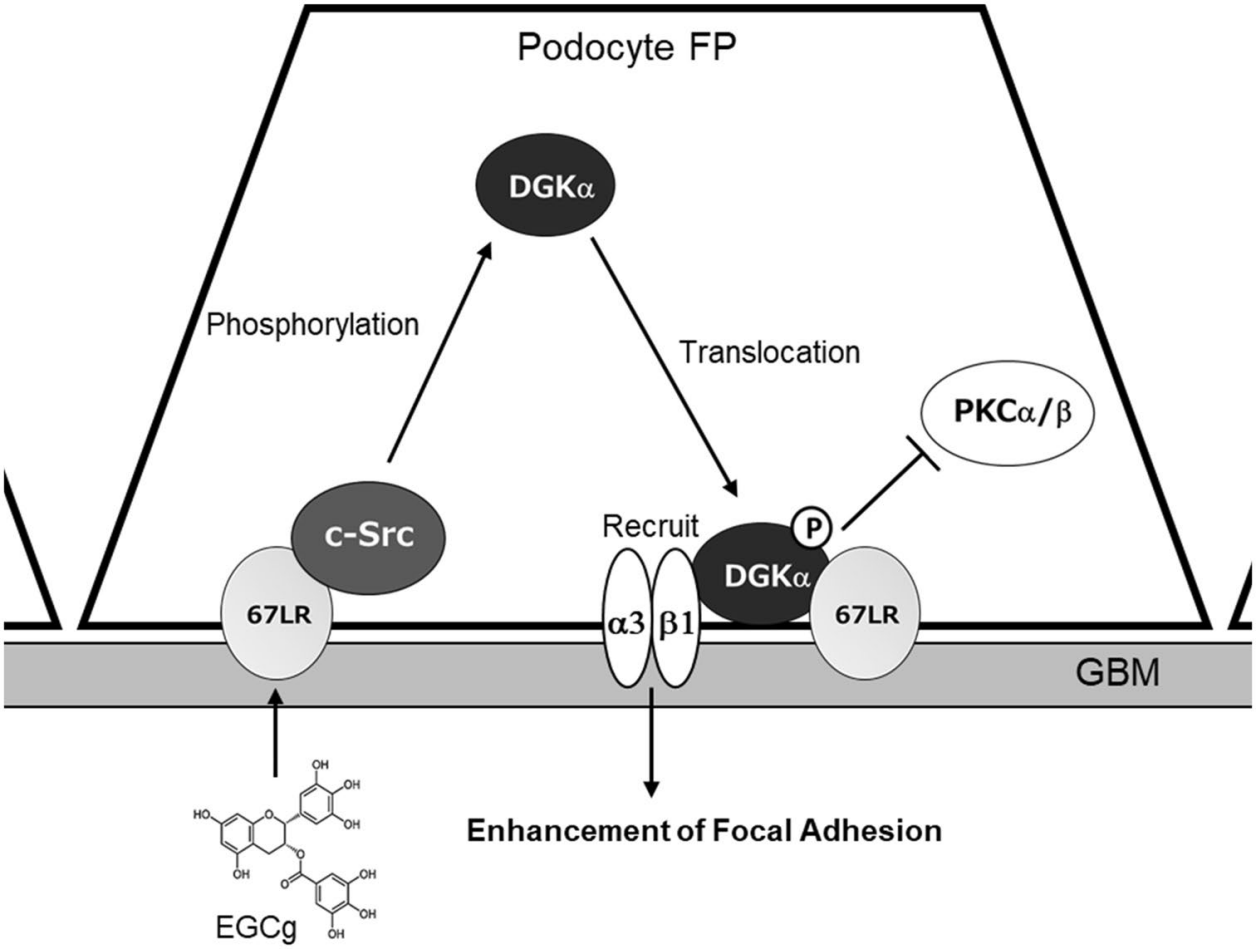
**The estimated mechanisms of DN amelioration by EGCg through DGKα activation in the podocyte foot process.** FP: Foot process, GBM: Glomerular basement membrane, α3: α3 integrin, β1: β1 integrin.

We previously reported that both DGKα and 67LR are expressed in podocytes^22^. As explained above, podocytes form a slit membrane structure, which functions as a filtration barrier by extending and interdigitating FPs. In other words, to maintain the glomerular filtration function, the morphology of the FP plays a pivotal role. To maintain the slit membrane structure, adhesion to neighboring FPs and the GBM is essential. Immunoelectron microscopy indicated that 67LR localized to the adhesive surface of the GBM, suggesting that activated DGKα translocated to the adhesive surface of the GBM and contributed to enhancing the adhesion between FPs and the GBM. Indeed, EGCg administration prevented the loss of podocytes in the DGKα^+/+^ mice but not in the DGKα^−/−^ mice. Additionally, paxillin staining clearly showed that EGCg significantly prevented a decrease in focal adhesion in the differentiated human podocytes. It is known that α3β1 integrin contributes to molecular adhesion to the GBM in podocytes and that DGKα recruits β1 integrin by producing PA^56–59^. Therefore, we hypothesized that DGKα activated by EGCg recruits β1 integrin to the adhesive surface of the GBM and enhances adhesion, which is another mechanism by which EGCg-induced activation of DGKα can ameliorate DN in addition to the inhibition of cPKCs (Fig. 6).

In this study, we used STZ-induced type 1 diabetic model mice because it was a well-established DN model and easy to make DGKα^−/−^ mice diabetic conditions^28^. However, it is known that STZ has direct toxicity on the kidneys^29,30^. Therefore, to justify the effect of EGCg on DN, we will take advantage of db/db or ob/ob type 2 diabetic model mice in the future study. We believe that EGCg should show the ameliorative effect of DN even in type 2 diabetes, considering the mechanisms of the effect of EGCg on DN.

It is well known that high blood pressure is one of the risk factors for the pathogenesis and progression of DN. It was reported that blood pressure is not affected by the STZ treatment^60,61^. Therefore, we assumed that there was little or no effect of blood pressure on the mice model used in the experiment. We evaluated albumin excretion and urine volume as outcomes of DN. However, as mentioned above, DN is diagnosed by the degree of kidney injury^1^. Since the STZ-induced diabetic mice model shows typical symptoms of DN, we assumed that EGCg ameliorated DN in this study. Indeed, we reported the amelioration of DN by αToc using the same STZ-induced diabetic mice^21,25^.

In summary, we revealed, for the first time, that DGKα is involved in the effect of EGCg on DN in vivo, and showed that, via activating DGKα, EGCg protected the loss of podocytes by preventing focal adhesion decreases. Moreover, this study revealed the mechanisms by which EGCg induces the amelioration of DN and implicates that DGKα is an attractive target for treating DN. To develop the medicine and/or functional food targeting on DGKα, as shown by the EGCg3″Me experiment, its absorption, stability, and efficiency to activate the enzyme is important.

## Supplementary information

Supplementary Information.
