# Supplementary material for: The mechanisms of ameliorating effect of a green tea polyphenol on diabetic nephropathy based on diacylglycerol kinase α
Source: Sci Rep. 2020 Jul 16;10:11790. doi: 10.1038/s41598-020-68716-6 (PMC7366667; doi:10.1038/s41598-020-68716-6)
Supplement: Supplementary file 1 — Supplementary Information. [file 41598_2020_68716_MOESM1_ESM.pdf]

Supplementary Information

The mechanisms of ameliorating effect of a green tea polyphenol on diabetic nephropathy based on diacylglycerol kinase α

Daiki Hayashi, Liuqing Wang, Shuji Ueda, Minoru Yamanoue, Hitoshi Ashida and Yasuhito Shirai*

Department of Applied Chemistry in Bioscience, Graduate School of Agricultural Science, Kobe University, Rokkodai-cho 1-1, Nada-ku, Kobe 657-8501, Japan

***Corresponding author:** Yasuhito Shirai, Department of Applied Chemistry in Bioscience, Graduate School of Agricultural Science, Kobe University, Rokkodai-cho 1-1, Nada-ku, Kobe 657-8501, Japan, TEL: +81-78-803-5889, Email: shirai@kobe-u.ac.jp

Supplementary Figures

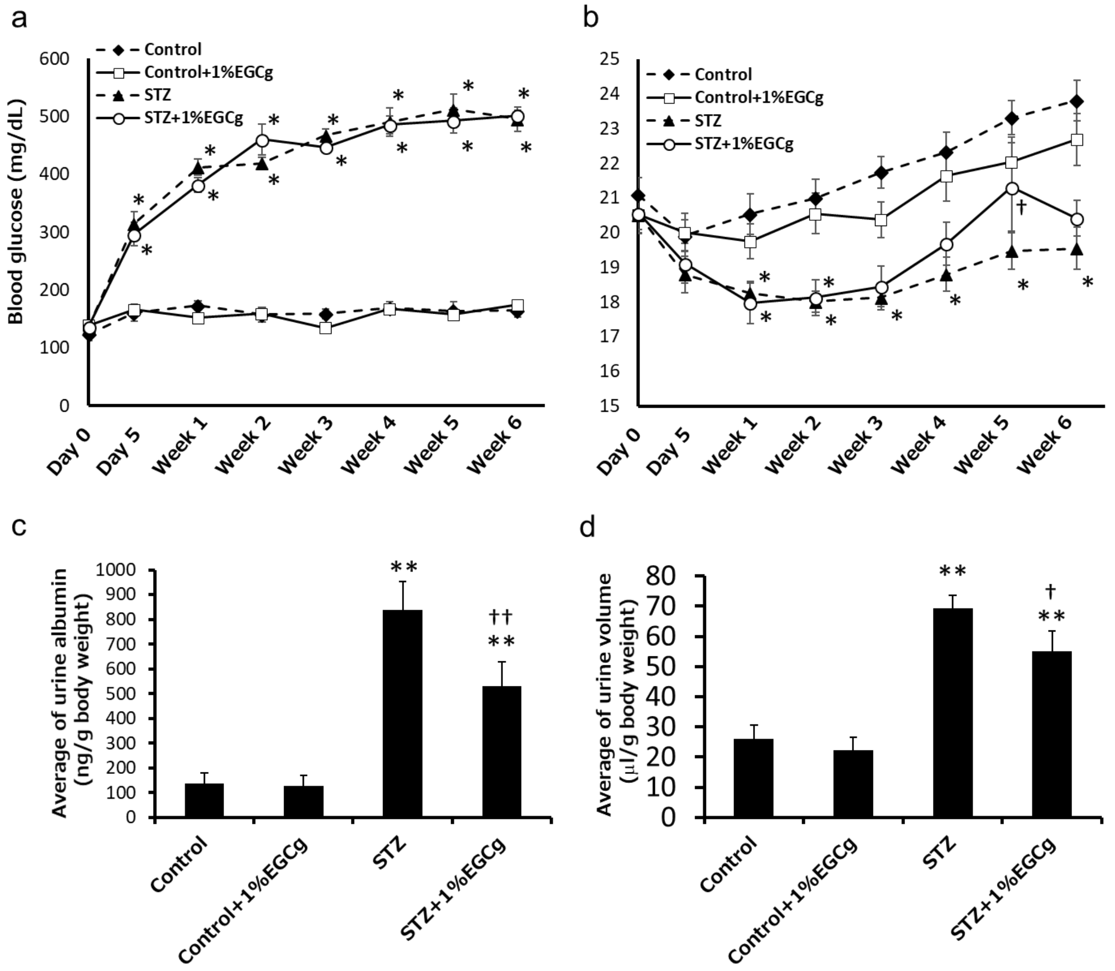

Supplemental Figure 1. The effects of oral administration of 1% EGCg on DN in mice. The blood glucose level (a) and bodyweight (b) of the mice in each group were measured before and after STZ administration (day 0 and day 5) and every week after that for 6 weeks. The average urine albumin amount (c) and urine volume (d) of mice from each group. The number of mice in every group was n=4~5. The values are means ± SE. *p < 0.05, *p<0.01 compared with respective control. † p<0.05 compared with STZ. One-way ANOVA followed by Tukey-Kramer's test between 4 groups was used for evaluating statistical significance.

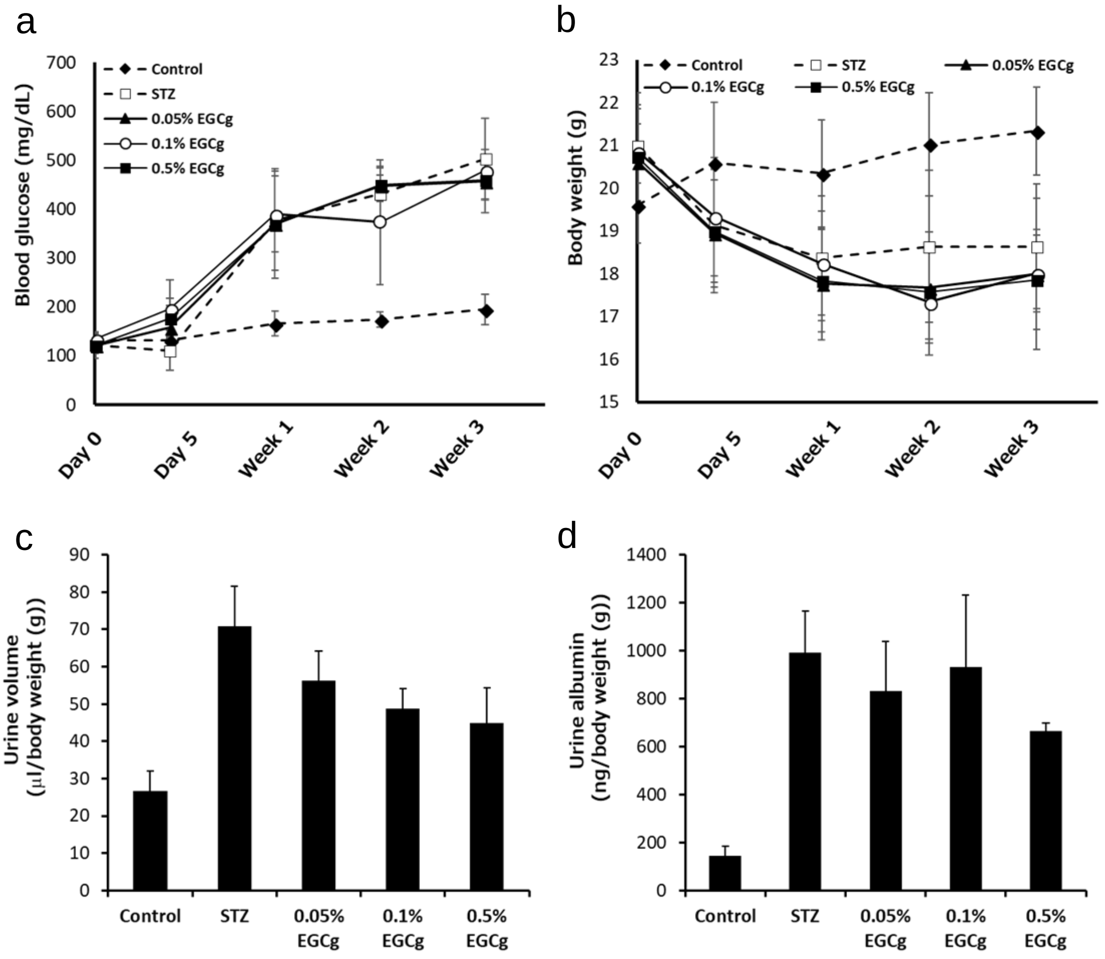

Supplemental Figure 2. Effect of various concentrations of EGCg on DN. Blood glucose level (a) and bodyweight (b) of mice were measured before/after STZ administration (Day 0 and 5) and every week until week 3. Urine albumin amount was measured by CBB staining of urine of mice from each group (c). Urine volume of mice from each group was measured by using metabolic case (d). The number of mice in every group was n=4~5. The values are means ± SE.

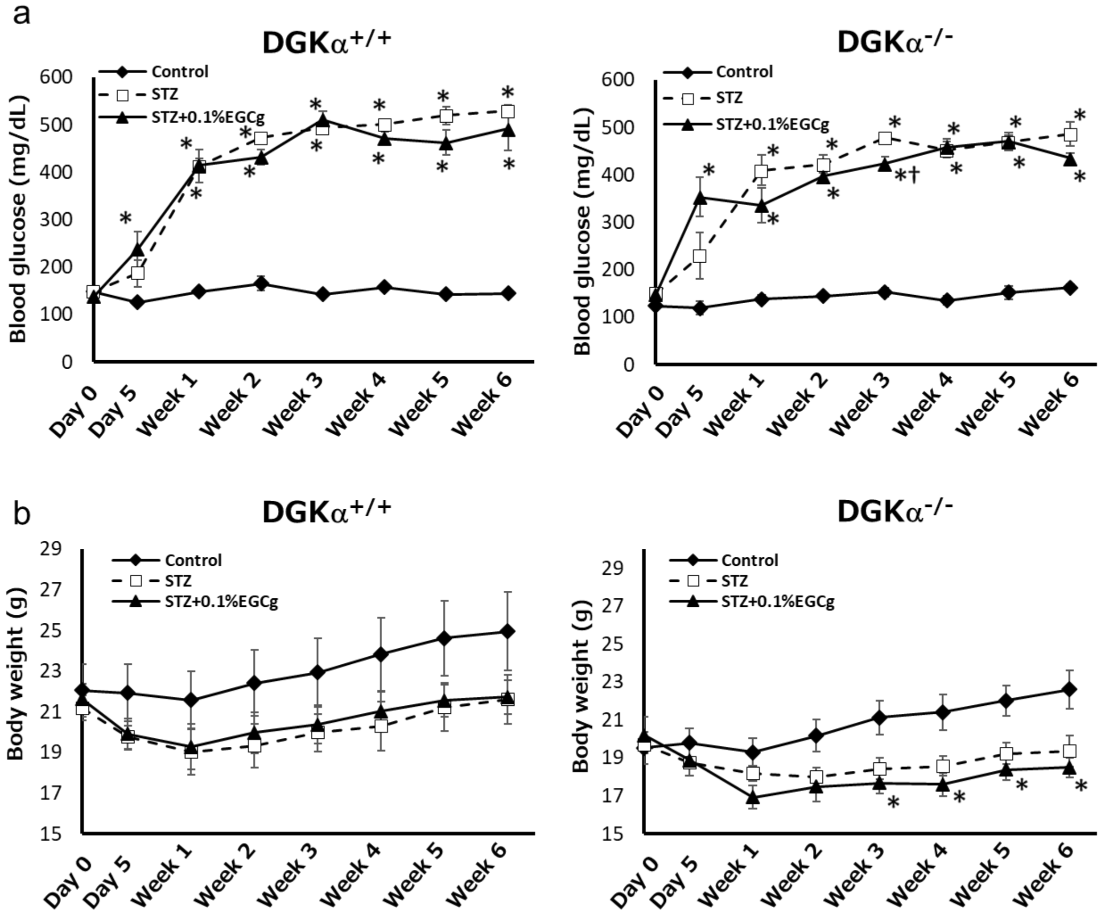

Supplemental Figure3. Effect of EGCg on blood glucose level and body weight of DGKα$^{+/+}$ and DGKα$^{-/-}$ mice. Blood glucose level (a) and body weight (b) of DGKα$^{+/+}$ and DGKα$^{-/-}$ mice were measured before/after STZ administration (Day 0 and 5) and every week until week 6. The number of mice in every group was n=4. The values are ±SE. *$p < 0.05$, compared with control. † $p<0.05$ compared with STZ. One-way ANOVA followed by Tukey-Kramer's test between 3 groups was used for evaluating statistical significance.

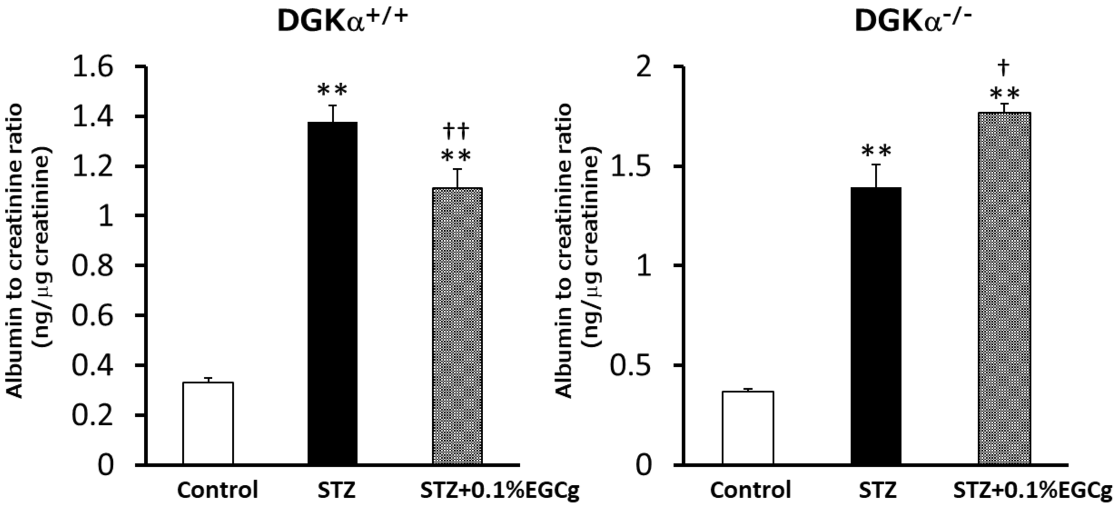

Supplemental Figure 4. The changes in the urine albumin to creatinine ratio.

The average urine albumin to creatinine ration of DGKα$^{+/+}$ and DGKα$^{-/-}$ mice. The number of mice in every group was n=4. The values are ±SE. *$p < 0.05$, *p<0.01 compared with control. †p<0.05, ††p<0.01 compared with STZ. One-way ANOVA followed by Tukey-Kramer's test between 3 groups was used for evaluating statistical significance.

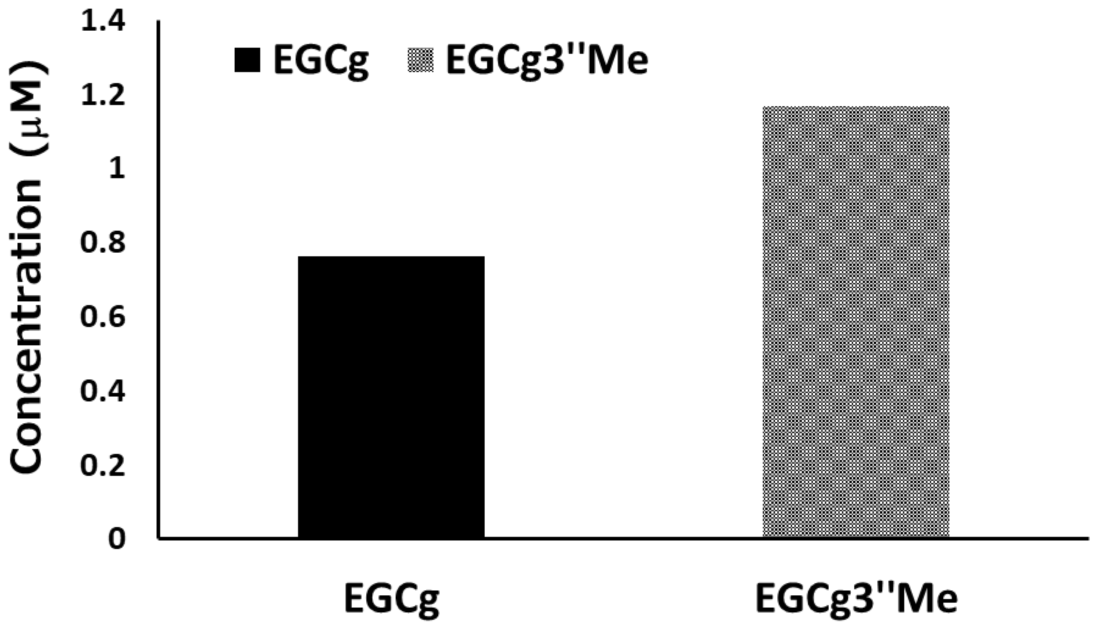

Supplemental Figure 5. The concentration of EGCg and EGCg3"Me in each group.

We collected plasma from mice fed with 0.05% EGCg or EGCg3"Me containing diet at the end of the experiment and measured the concentration of EGCg and EGCg3"Me by using HPLC. EGCg and EGCg3"Me were measured by absorption of light length 280 nm. The extraction method and conditions of HPLC were followed by the previous report (L. Wang et al., *Food Funct.,* 2018,**9**, 5362-5370).

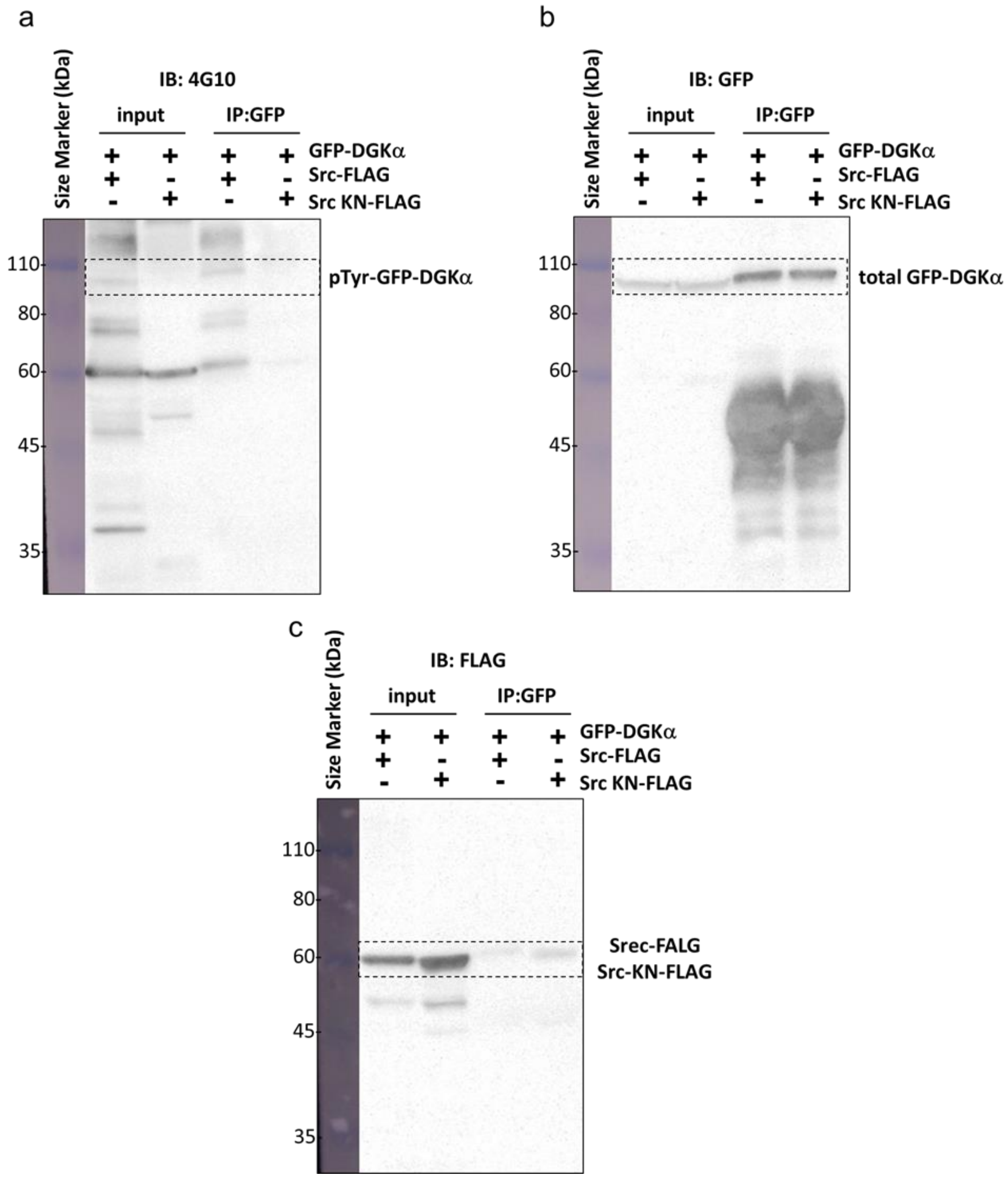

Supplemental Figure 6. Full-length image of the blotting for Figure 3b.
Dotted lines show trimmed areas for generating the figure.

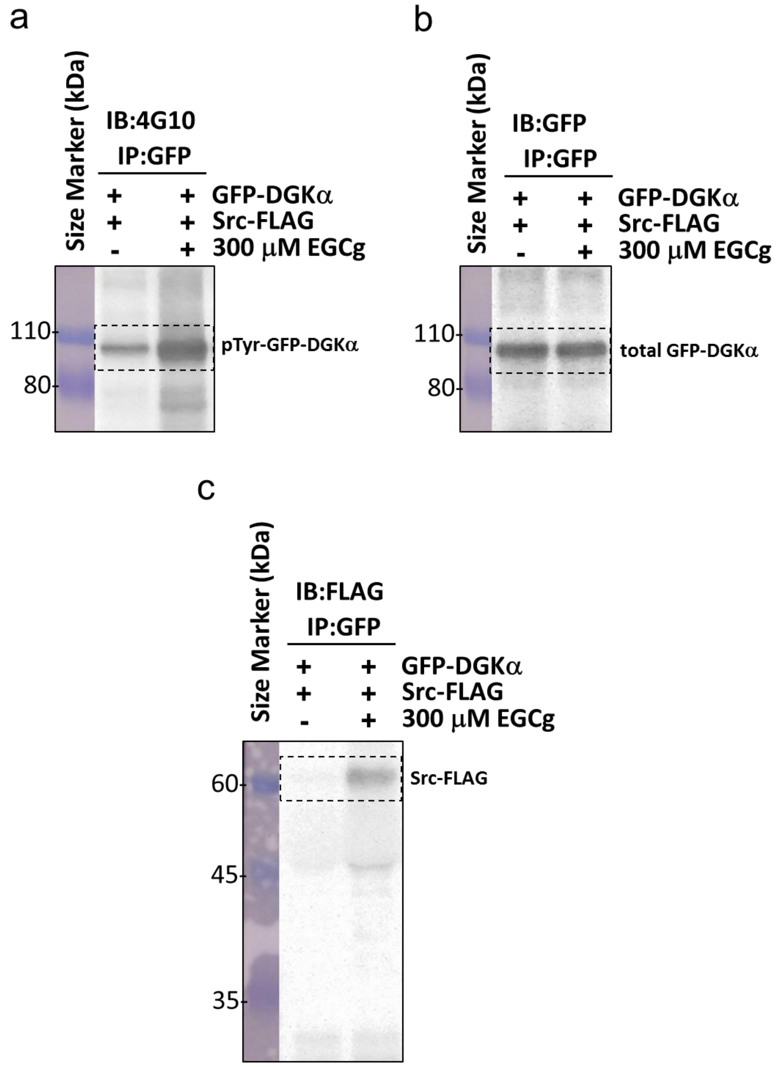

Supplemental Figure 7. Full-length image of the blotting for Figure 3c.

Dotted lines show trimmed areas for generating the figure.

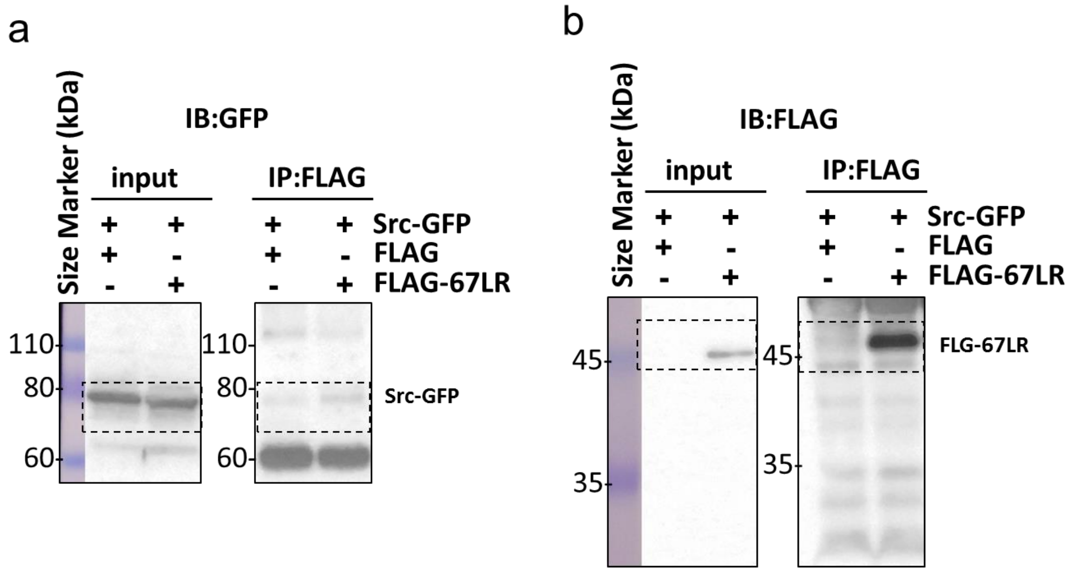

Supplemental Figure 8. Full-length image of the blotting for Figure 3e.
Dotted lines show trimmed areas for generating the figure.

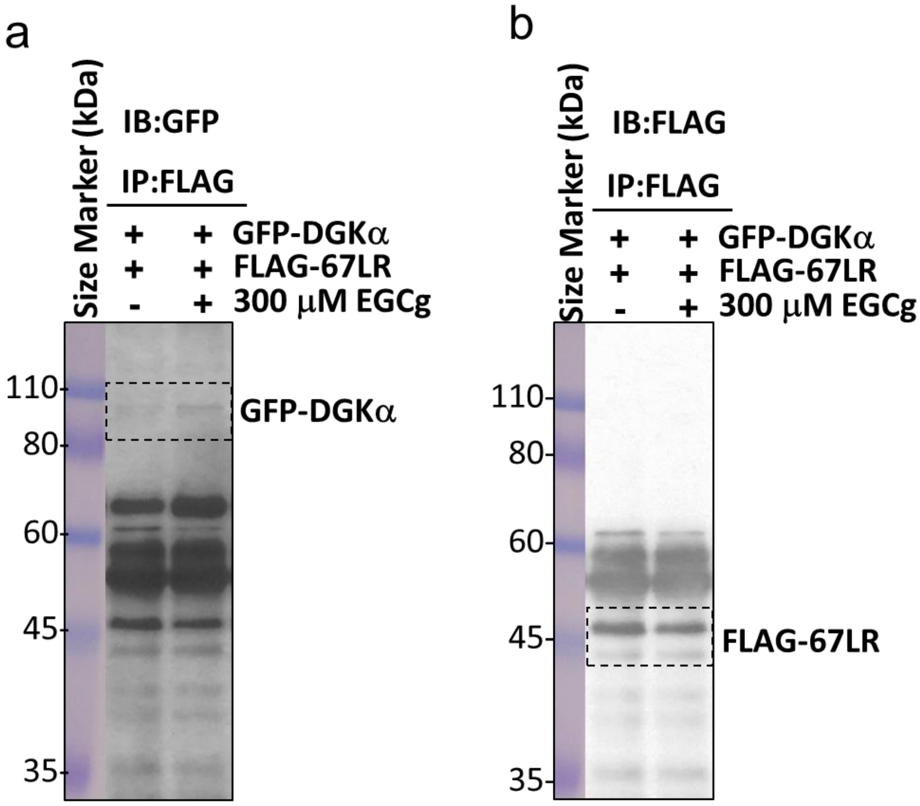

Supplemental Figure 9. Full-length image of the blotting for Figure 3g.
Dotted lines show trimmed areas for generating the figure.

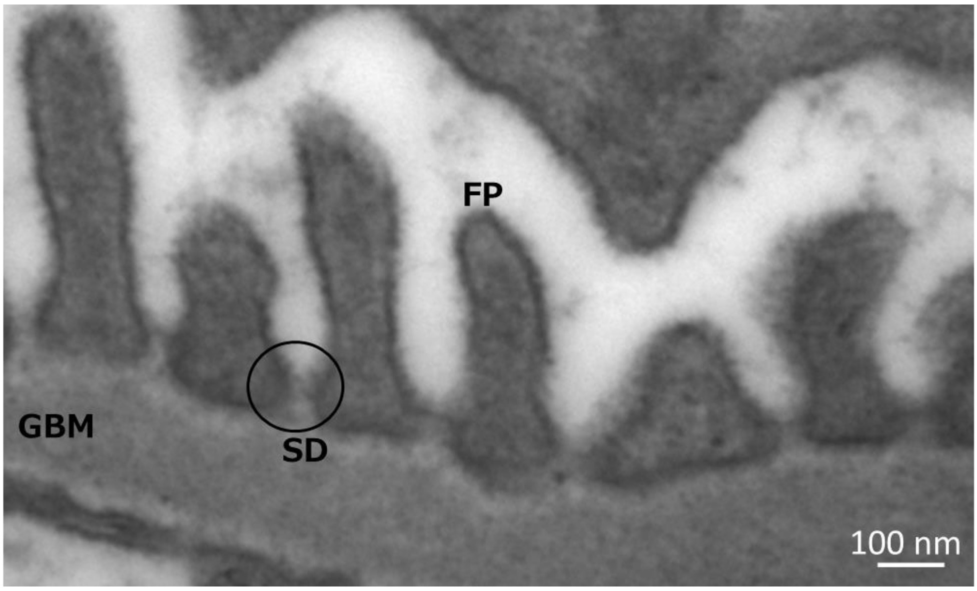

Supplemental Figure 10. The image of the slit membrane structure observed by transmission electron microscopy. FP: foot process, SD: slit diaphragm, GBM: glomerular basement membrane

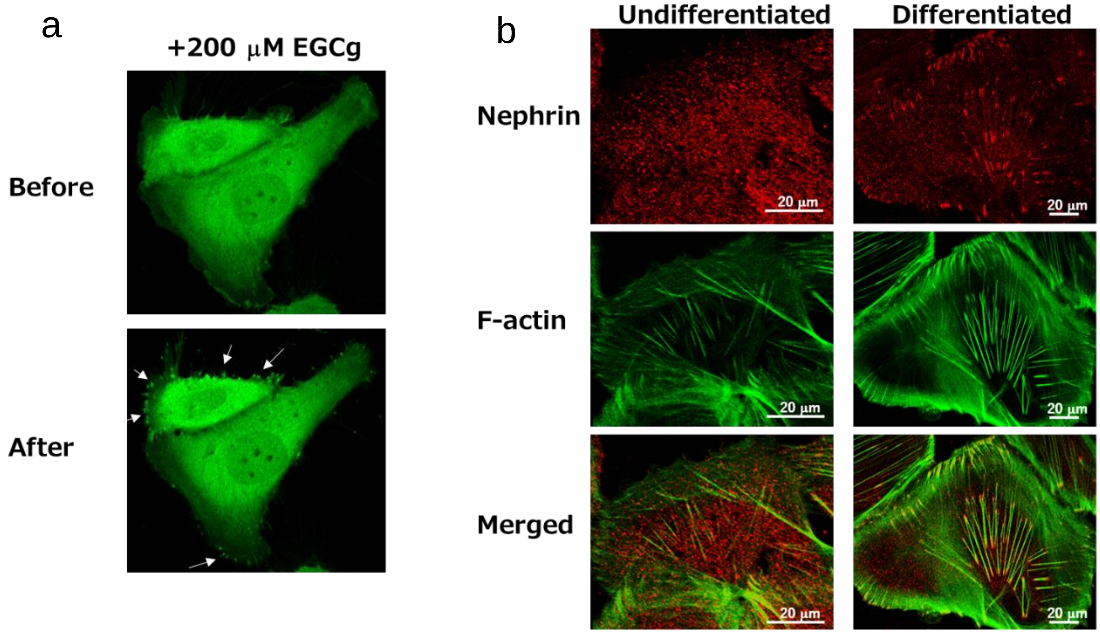

Supplemental Figure 11. Confirmation of GFP-DGKα translocation by EGCg, and differentiation of human immortalized podocyte. Typical images of GFP-DGKα in the human immortalized podocyte before and after stimulation with EGCg. GFP-DGKα was expressed in undifferentiated human immortalized podocyte and stimulated with EGCg at 200 μM under confocal laser scanning microscopy for 3 minutes (a). Arrows indicate the accumulation of GFP-DGKα on the plasma membrane. Typical images of stained nephrin and F-actin of undifferentiated and differentiated human immortalized podocyte (b).
